# Synergistic *in vitro* activity and mechanism of KBN lotion and miconazole nitrate against drug-resistant *Candida albicans* biofilms

**DOI:** 10.3389/fcimb.2024.1426791

**Published:** 2024-08-29

**Authors:** Xiaoyu Cao, Ni Xiao, Jingyi Huang, Li Li, Lian Zhong, Jun Zhang, Fengyun Wang

**Affiliations:** ^1^ School of Traditional Chinese Medicine, Guangdong Pharmaceutical University, Guangzhou, China; ^2^ School of Pharmaceutical Sciences, Guangzhou University of Chinese Medicine, Guangzhou, China

**Keywords:** *Candida albicans*, biofilms, KBN, miconazole nitrate, synergistic

## Abstract

**Background:**

In the face of increasing antifungal resistance among *Candida albicans* biofilms, this study explores the efficacy of a combined treatment using Kangbainian lotion (KBN) and miconazole nitrate (MN) to address this challenge.

**Methods:**

Using UPLC-Q-TOF/MS Analysis for Identification of Active Compounds in KBN Lotion; FICI for synergy evaluation, XTT and ROS assays for biofilm viability and oxidative stress, fluorescence and confocal laser scanning microscopy (CLSM) for structural and viability analysis, and real-time fluorescence for gene expression.

**Conclusion:**

Our study indicates that the combined application of KBN and MN somewhat impacts the structural integrity of *Candida albicans* biofilms and affects the expression of several key genes involved in biofilm formation, including *ALS1*, *ALS3*, *HWP1*, *HSP90*, and *CSH1*. These preliminary findings suggest that there may be a synergistic effect between KBN and MN, potentially influencing not only the structural aspects of fungal biofilms but also involving the modulation of genetic pathways during their formation.

## 1 Introduction

Vaginal vulvovaginal candidiasis (VVC) is a common and complex vaginal infection caused by fungi of the *Candida* genus, with symptoms influenced by host physiology, fungal biology, and immune responses, affecting the health and quality of life of millions of women worldwide. Among these, *C. albicans* is the primary pathogen of VVC. It invades the host and contributes to disease progression through various virulence factors (Tamura et al., 2007), such as adherence ability, biofilm formation, production of extracellular hydrolytic enzymes, hyphal morphogenesis, and phenotypic switching (Pereira et al., 2021).

The formation of biofilms is one of the major virulence factors of *C. albicans*. Unlike planktonic cells, *C. albicans* biofilms exhibit unique phenotypic characteristics, including significantly increased resistance to antifungal drugs, host defense mechanisms, and physical and chemical stress (Donlan and Costerton, 2002). The colonization and biofilm formation of *C. albicans* on vaginal mucosa is a key reason why conventional antifungal treatments are becoming increasingly difficult. These biofilms show growing resistance to widely used antifungal drugs, such as amphotericin B (Fernandes et al., 2015) and fluconazole (Taff et al., 2013), which may prevent the complete eradication of the pathogen from the vagina. This can lead to frequent recurrences of VVC and further progression of the disease.

In recent years, studies have shown that natural plants and their compounds, such as phenols, essential oils, terpenes, lectins, and alkaloids (Bersan et al., 2014; Fabri et al., 2021), may inhibit biofilm formation and disrupt mature biofilm structures of *C. albicans* by downregulating the expression of biofilm-related genes (Rodrigues de Araújo et al., 2019). Various natural compounds have also been found to enhance the antifungal effects of azole drugs. When fluconazole is combined with eugenol or cinnamaldehyde, its activity against *C. albicans* biofilms is enhanced, demonstrating synergistic effects (Khan and Ahmad, 2012; Pemmaraju et al., 2013). Further research has shown that thymol and menthol, when used in combination with fluconazole, exhibit synergistic inhibitory effects. These lipophilic compounds can penetrate cell membranes and disrupt the ergosterol biosynthesis pathway (Ahmad et al., 2011). Berberine has been found to exhibit a synergistic effect with miconazole against *C. albicans* biofilms (Wei et al., 2011). *Park* et al. further demonstrated that berberine enhances cell membrane permeability by inhibiting sterol 24-methyltransferase, a key enzyme in ergosterol biosynthesis (Park et al., 1999). Additionally, berberine can activate the Krebs cycle and inhibit ATP synthase activity, ultimately leading to oxidative cell damage through increased production of reactive oxygen species (ROS) (Ghannoum and Rice, 1999; Xu et al., 2009; Shi et al., 2010). *De Cremer* et al. screened a library of repurposed compounds to identify molecules that could enhance the effects of miconazole nitrate (MN), discovering three compounds—hexachlorophene, pyrvinium pamoate, and artesunate—that show synergistic effects with MN in treating mature *C. albicans* biofilms (De Cremer et al., 2015).

The plant-based preparation Kang Bai Nian (KBN) lotion has been shown to effectively inhibit the growth of susceptible *C. albicans in vitro*. Electron microscopy examinations have revealed that KBN can disrupt the organelles, cell membrane, and cell wall of susceptible *C. albicans* cells (Huang et al., 2020). In efficacy tests using a mouse model of VVC, KBN lotion was found to reduce the vaginal *C. albicans* load and inhibit hyphal growth (Chen et al., 2021). MN is a widely used clinical treatment for VVC, but the issue of increasing resistance during treatment is becoming more serious. Whether the combined use of MN and KBN lotion can combat resistant *C. albicans* by inhibiting the biofilm virulence factor warrants further in-depth research.

In this study, we focus on exploring the efficacy of KBN in combination with MN in inhibiting the biofilms of *C. albicans*. Through *in vitro* experiments and RT-PCR tests, we aim to preliminarily validate the potential mechanisms targeting biofilm-inhibiting genes, providing a foundational experimental basis for the future clinical use of KBN combined with MN in treating fungal infections.

## 2 Materials and methods

### 2.1 Preparation of KBN lotion

**Figure 1:**
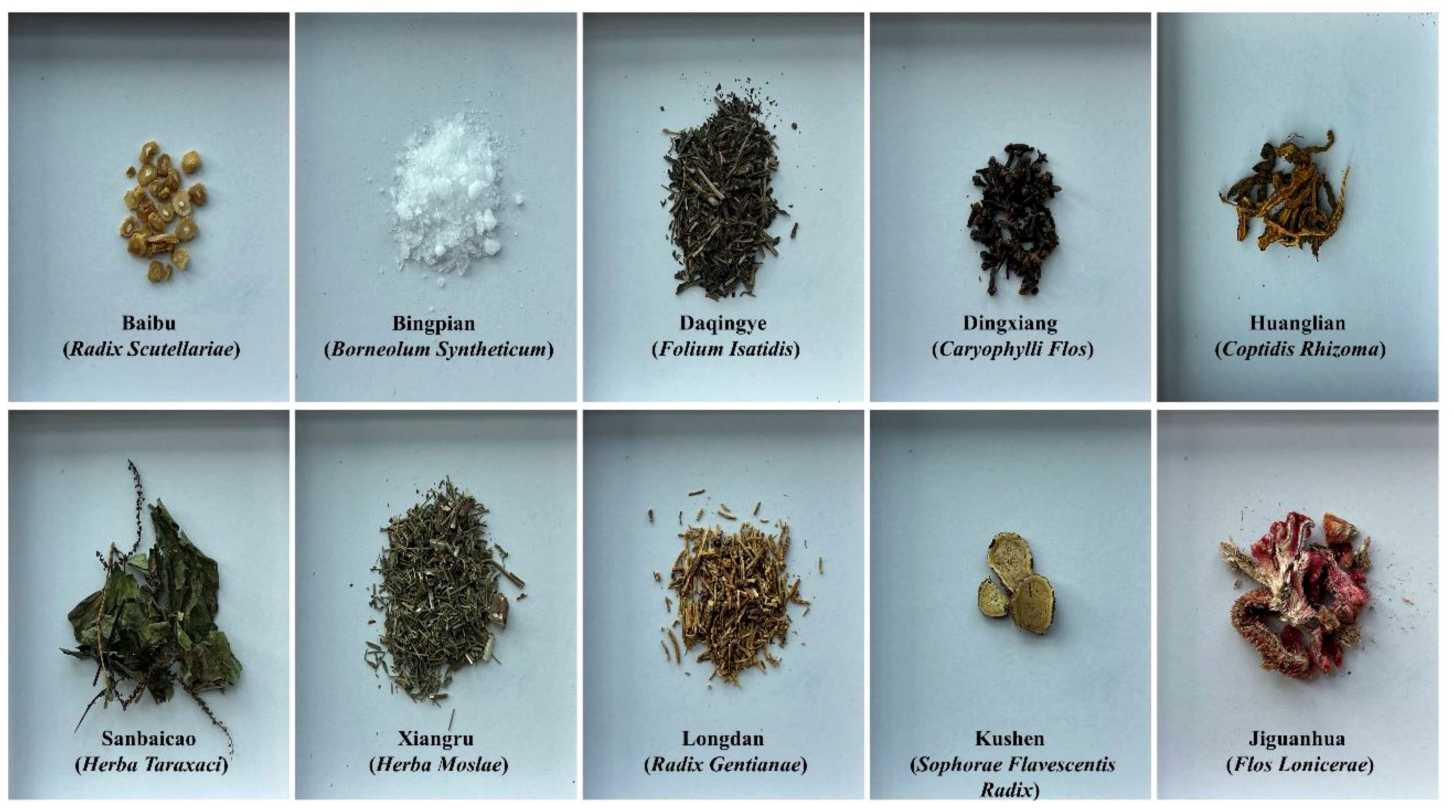
The voucher specimens of the plants used in the KBN lotion.

Take 75 g of Coptidis Rhizoma (Huanglian), 37.5 g of Herba Taraxaci (Sanbaicao), 20 g of Folium Isatidis (Daqingye), 20 g of Flos Lonicerae (Jiguanhua), 12.5 g of Herba Moslae (Xiangru), 12.5 g of Sophorae Flavescentis Radix (Kushen), 12.5 g of Radix Scutellariae (Baibu), 10 g of Radix Gentianae (Longdan), 5 g of Caryophylli Flos (Dingxiang), and 1 g of Borneolum Syntheticum (Bingpian), totaling 206 g. Except for Borneol, each herb was refluxed with 6 to 4 times the amount of 80% ethanol for 1 hour each time. The extract was filtered through a 200-mesh sieve and the filtrate was combined. Concentrate under reduced pressure to obtain a concentration of 1.0 g crude drug/mL. Dissolve 1 g of Borneo in a small amount of ethanol, then add it to the mixture and mix thoroughly to obtain KBN lotion. Borneol was purchased from Yunnan Linyuan Spice Co., Ltd. (Yunnan, China), the rest of the botanical drugs were purchased from Guangzhou Zhixin Chinese Medicine Pieces Co., Ltd. (Guangzhou, China) (**Figure 1**).

### 2.2 UPLC-Q-TOF/MS

Weigh 0.50 g of KBN lotion and ultrasonically extract it in 10 ml of 50% methanol-water for 1 hour. Subsequently, filter out the residue. Centrifuge the extract at 4°C and 12,000 rpm for 15 minutes, then filter the supernatant through a 0.22 μm membrane. Dilute the filtrate 5 times before performing UPLC-Q-TOF/MS (SCIEX TripleTOF^®^ 6600+ LC-MS/MS, USA) analysis. The composition of the two mobile phases was 0.1% (v/v) formic acid in water (A) and acetonitrile (B): 0–15 min, 5%–35% B; 15–20 min, 35%–90% B; 20–28 min, 90%–5% B; 28–34 min, 5% B. The separations were performed with a constant flow rate of 0.3 ml/min. Scanning Mode: +ESI and -ESI; Acquisition Range: Mass 50-1500 M/Z. Capillary Voltage ISVF: 5500V; Cone Voltage CE: ± 35V; Ion Source Temperature: 550°C. Cone Gas Flow: 50 L/H. Injection Volume: 1 µL.

### 2.3 Preparation of activated strains and fungal suspensions of *C. albicans*

Inoculate white *C. albicans* on Sabouraud Dextrose Agar medium (SDA) and incubate in a constant temperature incubator at 35°C for 24 hours. Pick a single colony (with a diameter greater than 1mm) and inoculate it onto Yeast Extract Peptone Dextrose medium (YEPD). Adjust the yeast concentration using a McFarland turbidity tube (Huankai, China), then incubate on a constant temperature shaker at 30°C with shaking at 200 rpm for 16 hours to reach the logarithmic growth phase. Use a hemocytometer for counting and adjust the concentration of the bacterial suspension to 1*10^3^CFU/ml with RPMI1640 liquid medium(Gibco, USA). In this study, the *C. albicans* quality control strain CMSS(F)98001 was obtained from the National Institutes for Food and Drug Control (NIFDC) of China. Additionally, the clinically isolated drug-resistant strains *C. albicans 901*, *904*, *953*, and *311* were generously provided by Professor Yuanying Jiang from the Second Military Medical University.

### 2.4 Minimum inhibitory concentration test

**Figure 2:**
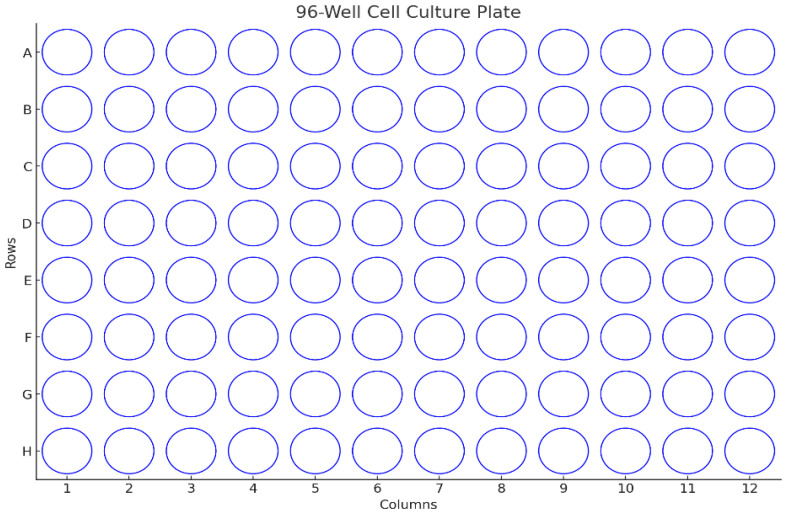
The schematic diagram of a 96-well cell culture plate.

The antifungal susceptibility test for KBN lotion against *C. albicans* was conducted using the microbroth dilution method according to the M27-A3 standard protocol recommended by the Clinical and Laboratory Standards Institute (CLSI) of the United States ((CLSI), C. a. L. S. I, 2008). A sterile 96-well cell culture plate was used for the assay (**Figure 2**). In each row, 100 µL of blank RPMI 1640 liquid medium was added to the first well to serve as a blank control. Wells 3 to 12 in each row were inoculated with 100 µL of a fungal suspension. The second well in each row received a mixture of x µL of the detergent stock solution and (200-x) µL of the *C. albicans* suspension. A two-fold serial dilution was performed from the second to the eleventh well in each row using a multichannel pipette, and then 100 µL of liquid was removed from the eleventh well. This procedure was repeated three times. The 96-well plate was incubated at 37°C for 48 hours in a constant temperature incubator. Optical density (OD) values at 630 nm were measured using a microplate reader (BioTek, Winooski, Vermont, USA).

### 2.5 Fractional inhibitory concentration index

To prepare the experimental setup, a sterile 96-well cell culture plate was used (**Figure 2**). The first well of each row was filled with 100 µL of RPMI 1640 liquid medium to serve as a blank control. In well A2, 2x µL of KBN lotion was combined with (200-2x) µL of the fungal suspension. For wells A3 to A11, (200-x) µL of the fungal suspension was mixed with x µL of KBN lotion. The remaining wells were inoculated with 100 µL of the fungal suspension. A two-fold serial dilution was performed from wells A2 to A11 down to wells F2 to F11 using a pipette, and 100 µL of liquid was removed from wells F2 to F11. In wells A2 to G2, y µL of miconazole nitrate solution was added along with (100-y) µL of the fungal suspension. In well H2, z µL of KBN lotion was added along with (100-z) µL of the fungal suspension. Another two-fold serial dilution was performed from well 2 to well 11 in each row, with 100 µL of liquid removed from well 11 in each row. The susceptibility plate was incubated at 37°C for 48 hours, and the results were interpreted as previously described. The experiment was repeated three times. The evaluation of drug combination effects was conducted using the Fractional Inhibitory Concentration Index (FICI) model.

### 2.6 XTT assay

Add 100 µL of fungal suspension to a sterile 96-well cell culture plate and incubate at 37°C for 1.5 hours (for early biofilm formation) or 24 hours (for mature biofilm) to allow *C. albicans* to adhere. After adhesion, aspirate the medium and rinse twice with PBS buffer. Then, add 100 µL of the following treatments: KBN 6.25 mg/mL, KBN 0.78 mg/mL + MN 0.25 µg/mL, KBN 0.39 mg/mL + MN 0.25 µg/mL, KBN 0.19 mg/mL + MN 0.25 µg/mL, MN 4 µg/mL, and a blank control of RPMI 1640 liquid medium. Incubate statically in a constant temperature incubator at 37°C for 24 hours. Afterwards, aspirate the supernatant and rinse twice with PBS buffer. In the dark, add 200 µL of XTT/menadione solution (sigma, USA) to each well, cover with aluminum foil to protect from light, and incubate statically in a constant temperature incubator at 37°C for 1 hour. Then, transfer 100 µL of the supernatant to a new sterile 96-well cell culture plate and measure the OD value at 492 nm to calculate the biofilm formation inhibition rate (Koban et al., 2012).

### 2.7 ROS assay

Quantitative determination of intracellular reactive oxygen species levels using the oxidation-sensitive DCFH-DA (sigma, USA) dye. Add 100 µL of fungal suspension to a sterile black 96-well cell culture plate and incubate at 37°C for 1.5 hours (for early biofilm formation), 24 hours (for mature biofilm formation), and 48 hours (for late-stage mature biofilm formation) to allow *C. albicans* to adhere. After adhesion, aspirate the culture medium and rinse twice with sterile PBS buffer. Then, add 100 µL of the following treatments: KBN at 6.25 mg/mL, KBN at 0.78 mg/mL + MN at 0.25 µg/mL, KBN at 0.39 mg/mL + MN at 0.25 µg/mL, KBN at 0.19 mg/mL + MN at 0.25 µg/mL, MN at 4 µg/mL, and a blank control of RPMI 1640 medium. Incubate statically at 37°C for 24 hours, then add DCFH-DA dye (10 µM, 30 µL) and incubate in the dark at 37°C for 40 minutes. Remove the fungal suspension and wash twice with sterile PBS buffer to remove any DCFH-DA dye not internalized by the cells, then re-add 200 µL of sterile RPMI 1640 liquid medium. Finally, measure fluorescence intensity under ex-citation at 488 nm and emission at 522 nm.

### 2.8 Fluorescence microscopy

Sterile 1 cm × 1 cm cover slips are soaked overnight in fetal bovine serum and prepared for use. Place the cover slips in a sterile 24-well cell culture plate, adding 1 mL of fungal suspension to each well, and incubate at 37°C for 1.5 hours, 24 hours, and 48 hours to form early, mature, and late-stage biofilms of *C. albicans*, respectively. Wash three times with sterile PBS to remove planktonic cells. For the experimental groups, add 0.5 mL of the following treatments to each well: KBN 6.25 mg/mL, KBN 0.78 mg/mL + MN 0.25 µg/mL, KBN 0.39 mg/mL + MN 0.25 µg/mL, KBN 0.19 mg/mL + MN 0.25 µg/mL, and MN 4 µg/mL. The blank control group receives 0.5 mL of blank RPMI 1640 liquid medium. Continue to incubate at 37°C for 24 hours, then wash three times with sterile PBS to remove planktonic cells. Add 20 μL of FITC-conA (sigma, USA) to each well and stain in the dark at room temperature for 1 hour, followed by three washes with cold PBS. Observe under a fluorescence microscope(Leica DM2500 LED Optical microscope, Germany).

### 2.9 CLSM

Use sterile cell culture dishes, adding 2 mL of fungal suspension to each dish, and incubate at 37°C for 1.5 hours, 24 hours, and 48 hours to form early-stage and mature biofilms of *C. albicans*, respectively. Wash three times with cold sterile PBS to remove planktonic cells. To each dish, add 2 mL of the following treatments: KBN 6.25 mg/mL, KBN 0.78 mg/mL + MN 0.25 µg/mL, KBN 0.39 mg/mL + MN 0.25 µg/mL, KBN 0.19 mg/mL + MN 0.25 µg/mL, and MN 4 µg/mL. The blank control group receives 2 mL of blank RPMI 1640 liquid medium. Continue incubating at 37°C for 24 hours, then wash three times with sterile PBS to remove planktonic cells. Following the instructions of the SYTO 9/PI Live/Dead Bacterial Double Stain Kit(MK, shanghai, China), add 1.5 µL of SYTO-9 and 1.5 µL of PI to each culture dish (Pakkulnan et al., 2019; Yu et al., 2022). Incubate in the dark at room temperature for 30 minutes and observe by CLSM (TCS SP8, Leica, Germany).

### 2.10 RT-PCR

Use a sterile, enzyme-free 6-well cell culture plate, adding 2 mL of fungal suspension to each well, and incubate at 37°C for 1.5 hours, 24 hours, and 48 hours to form early-stage and mature biofilms of *C. albicans*, respectively. Wash three times with cold sterile PBS to remove planktonic cells. To each well, add 2 mL of the following treatments: KBN 6.25 mg/mL, KBN 0.78 mg/mL + MN 0.25 µg/mL, KBN 0.39 mg/mL + MN 0.25 µg/mL, KBN 0.19 mg/mL + MN 0.25 µg/mL, and MN 4 µg/mL. The blank control group receives 2 mL of blank RPMI 1640 liquid medium. After continuing the incubation for 24 hours, use a sterile, enzyme-free pipette tip to aspirate the culture medium, then scrape off the biofilms adhered to the wells with a biofilm scraper, collect the scraped cells, centrifuge to remove the supernatant, wash once with DEPC water, centrifuge again and discard the supernatant for later use. Total RNA of each group was extracted by Trizol method. The RNA sample was reverse transcribed into cDNA according to the instructions of PrimeScript™ One Step RT-PCR Kit Ver.2 (Takara Bio Inc.). For the design of primers, refer to **Table 1**.

**Table 1:** Specific primer sequence and PCR product length.

Target gene	Target gene sequence(5’-3’)	Product
ALS1 F	GTGGATCTGTTACTGGTGGAGC	154bp
ALS1 R	ATGAATGTGTTGGTTGAAGGTGA	
ALS3 F	GAGTGGAAGCAGCTGTGGAAG	140bp
ALS3 R	TGTTCCAACAACTGAAAGTGAGG	
HWP1 F	TTTCTACTGCTCCAGCCACTG	118bp
HWP1 R	ACTTCAGATTCGGTACAAGAGCT	
HSP90 F	GACCGTTAAGGACTTGACCACT	116bp
HSP90 R	ATCCCAAGGCAATCAATCTGT	
CSH1 F	GGTTCCGTACTTTCGATACTGCT	154bp
CSH1 R	GAACTGTCTTCTGCGTCGTCT	
18S RNA-F	ATTGCGATAACGAACGAGACC	110bp
18S RNA-R	TGCCTCAAACTTCCATCGACT	

### 2.11 Statistical analysis

RT-PCR data were expressed by ^-^x± S, and statistical analysis was performed using SPSS 21.0. The data were first tested for normal distribution. If they conformed to a normal distribution, a homogeneity of variance test was performed. When variances were homogeneous and sample sizes were equal across groups, pairwise comparisons were conducted using Tukey’s test in one-way ANOVA. When variances were homogeneous but sample sizes were unequal, Scheffé’s test was used for pairwise comparisons. If variances were not homogeneous, Dunnett’s T3 test in one-way ANOVA was employed for pairwise group comparisons. A significance level of P<0.05 was considered indicative of statistical differences. Statistical analyses were conducted using GraphPad Prism 8.0. All experiments were triplicated and repeated three times on different days.

## 3 Results

### 2.1 UPLC-Q-TOF/MS

**Figure 3:**
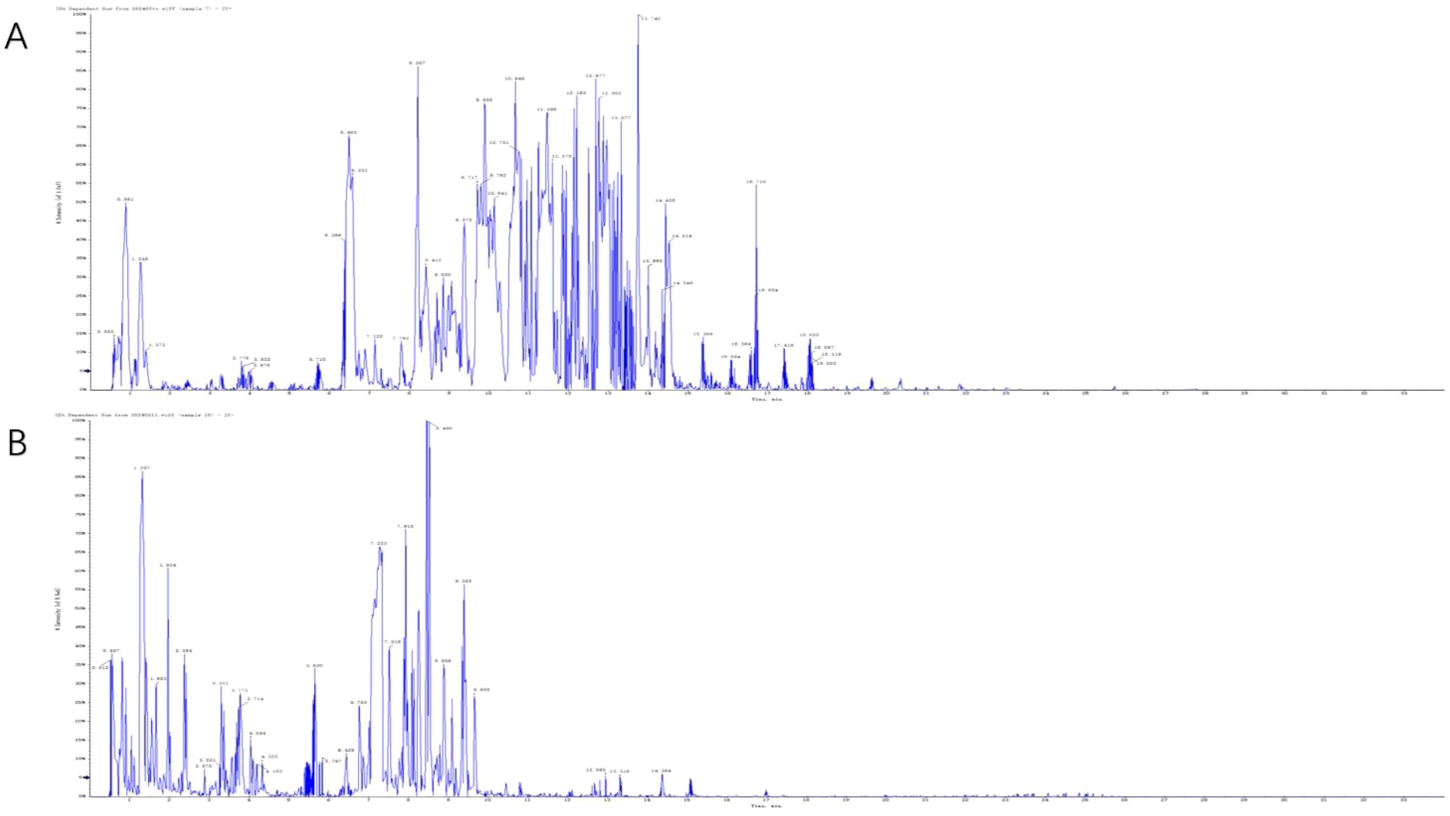
The total ion chromatogram obtained in the positive **(A)** and negative **(B)** modes.

The active compounds in KBN lotion were identified using UPLC-Q-TOF/MS analysis, which also provided the total ion chromatograms (**Figures 3A, B**). Information on the chemical constituents of KBN lotion was collected and organized by searching databases such as CNKI, Medline, PubMed, and NIST. A total of 44 compounds were identified, including 16 alkaloids, 13 flavonoids, 4 phenols, 2 essential oils, 2 caffeoylquinic acids, 1 organic acid, 1 lignan, 1 tannin, 1 nucleosides, and 1 sugar (**Table 2**). According to the results of UPLC/Q-TOF MS, the main components of KBN lotion are alkaloids and flavonoids, many of which exhibit antibacterial activity.

**Table 2:** Components of KBN lotion.

No	RT (min)	Extracting ions	Measured mass (m/z)	Formula	Identity	Error (ppm)	Main secondary fragment ions (MS/MS)	Category	Cas no
1	0.6241333	[M-H]-	341.108	C12H22O11	Sucrose	-2.8	179,89,119	Sugar	57-50-1
2	0.7882167	[M+H]+	265.1921	C15H24N2O2	13a-Hydroxylupanin	3.8	148,150,247	Alkaloids	15358-48-2
3	0.9240333	[M+H]+	247.1801	C15H22N2O	Sophocarpine	-1.6	245,179,136	Alkaloids	6483-15-4
4	1.018617	[M-H]-	169.0095	C7H6O5	Gallic acid	-26.6	125	Tannins	149-91-7
5	1.132767	[M-H]-	133.011	C4H6O5	Malic acid	-22.6	115,71	Organic acids	6915-15-7
6	1.17335	[M+H]+	249.1974	C15H24N2O	Matrine	5.2	148,150	Alkaloids	519-02-8
7	1.264683	[M+H]+	265.1938	C15H24N2O2	Oxymatrine	14.3	247,205,148,136	Alkaloids	16837-52-8
8	1.4005	[M+H]+	268.1068	C10H13N5O4	Adenosine	10.4	136,119	Nucleosides	58-61-7
9	1.430583	[M-H]-	151.0361	C8H8O3	4-hydroxyphenylacetic acid	-20.5	123,107	Phenols	156-38-7
10	2.37285	[M-H]-	153.0151	C7H6O4	Protocatechuic acid	-27.6	109,108	Phenols	99-50-3
11c	3.809183	[M+H]+	377.1431	C16H24O10	loganic acid	8.2	213,169	Terpenes	22255-40-9
12a	4.73045/14.24/17.023	[M+H]+	165.0929	C10H12O2	Isoeugenol	11.5	91,77	Essential oils	97-54-1
13	5.308917	[M+H]+	338.1981	C18H27NO5	platyphylline	-5.6	236,264	Alkaloids	480-78-4
14a	5.707/6.88765	[M+H]+	355.1013	C16H18O9	Chlorogenic Acid	-3.0	235,205,319	Caffeoylquinic Acids	327-97-9
15ac	5.716383/6.89	[M+H]+	355.1042	C16H18O9	chlorogenic acid	3.2	235,205,259,319	Caffeoylquinic Acids	327-97-9
16	6.530334	[M+H]+	342.1727	C20H23NO4	Isocorydine	7.9	279,265,237	Alkaloids	475-67-2
17	6.858217	[M-H]-	137.0204	C7H6O3	Salicylic acid	-29.3	93	Phenols	69-72-7
18	7.454433	[M+H]+	195.0656	C10H10O4	Vanillin acetate	2.1	77,91,103	Essential oils	881-68-5
19	7.800583	[M+Na]+	379.1007	C16H20O9	Gentiopicrin	1.8	158,200,217	Terpenes	20831-76-9
20	7.91575	[M+Na]+	349.1379	C20H22O4	Licarin A	-6.0	137,272,314	Lignans	51020-86-1
21	8.696016	[M+H]+	625.1718	C28H32O16	Isorhamnetin 3-glucoside-7-rhamnoside	2.9	343,445,463,313	Flavonoids	17331-71-4
22	8.90575	[M-H]-	359.0692	C18H16O8	Rosmarinic acid	-22.4	161,197,179	Phenols	20283-92-5
23	9.063	[M+H]+	340.1571	C20H21NO4	Papaverine	8.1	324,309	Alkaloids	58-74-2
24	9.154333	[M+H]+	187.0883	C11H10N2O	Deoxyvasicinone	9.1	187,118,120	Alkaloids	530-53-0
25	9.40765	[M+NH4]+	372.1823	C21H22O5	Xanthohumol	4.7	222,189,161	Flavonoids	6754-58-1
26	9.545466	[M+H]+	287.056	C15H10O6	Kaempferol	3.5	153,165,213	Flavonoids	520-18-3
27	9.6158	[M+H]+	285.0789	C16H12O5	Calycosin	-3.8	270,168,140	Flavonoids	20575-57-9
28	9.639133	[M]+	337.1278	C20H18NO4	Berberine	-9.1	321,292,278	Alkaloids	2086-83-1
29	10.0696	[M+H]+	324.1256	C19H17NO4	Stylopine	7.9	309,294,266	Alkaloids	84-39-9
30a	10.086/16.7185	[M+H]+	352.1183	C20H17NO5	Oxoglaucine	0.8	322,337,294,308	Alkaloids	5574-24-3
31a	10.0921/16.707	[M+H]+	352.1199	C20H17NO5	Oxyberberine	5.5	336,308,322,294	Alkaloids	19716-60-0
32	10.15977	[M+H]+	338.1374	C20H19NO4	Dihydroberberine	-3.8	322,307,306	Alkaloids	483-15-8
33	10.15977	[M+Na]+	397.1128	C16H22O10	Swertiamarin	7.1	301,235,205	Alkaloids	17388-39-5
34	10.18243	[M+H]+	354.1724	C21H23NO4	Dihydropalmatine	6.7	338,323	Alkaloids	26067-60-7
35	10.48223	[M+H]+	303.0499	C15H10O7	Quercetin	-0.1	229,153	Flavonoids	117-39-5
36	10.90972	[M+H]+	317.0668	C16H12O7	Isorhamnetin	3.8	302,153,170	Flavonoids	480-19-3
37	14.0955	[M+H]+	285.0705	C16H12O5	Maackiain	1.7	270,168	Flavonoids	2035-15-6
38	14.68963	[M+H]+	269.0817	C16H12O4	Formononetine	-1.4	197,253,213,237	Flavonoids	485-72-3
39	15.38308	[M+H]+	285.0786	C16H12O5	Wogonin	10.0	168,140	Flavonoids	632-85-9
40	16.5545	[M+H]+	263.0821	C16H10N2O2	Indirubin	0.4	235,219,206,132	Alkaloids	479-41-4
41	16.7185	[M+H]+	455.2128	C26H30O7	Kushenol I	6.2	179,303,153, 285,313	Flavonoids	99119-69-4
42	17.023333	[M+H]+	165.0913	C10H12O2	Eugenol	1.8	109,124,137,81	Flavonoids	97-53-0
43	18.98185	[M+H]+	453.225	C27H32O6	2’-O-methyl-Kurarinone	11.0	179,329,303	Flavonoids	270249-38-2
44	21.89415	[M+H]+	439.209	C26H30O6	Kurarinone	-2.3	179,303	Flavonoids	34981-26-5

### 2.2 MICs of KBN and MN against *C. albicans*

As shown in **Table 3**, the results of the *in vitro* antifungal drug susceptibility test show that the MICs of Fluconazole (FLC), Ketoconazole (KET), and MN against the quality control sensitive *C. albicans* 98001 are 1 µg/mL, <0.015 µg/mL, and <0.12 µg/mL, respectively. Against resistant *C. albicans* strains (901, 904, 311), the MICs are >64 µg/mL, >8 µg/mL, and 2 µg/mL or 1 µg/mL, respectively (**Table 3**). The results suggest that the resistant *C. albicans* strains (901, 904, 311) are resistant to Fluconazole and Ketoconazole, consistent with the standards recommended by the Clinical and Laboratory Standards Institute (CLSI) in the M27-A3 protocol.

**Table 3:** The MIC of KBN lotion and MN against *C. albicans*.

*C. albicans* Strains	KBN(mg/mL)	MN(μg/ml)	KET(μg/ml)	FLC(μg/ml)
**98001**	6.25	<0.12	<0.015	1
**901**	6.25	2	>8	>64
**904**	6.25	2	>8	>64
**953**	6.25	2	>8	>64
**311**	6.25	2	>8	>64

The *in vitro* antifungal drug susceptibility test results indicate that, for both sensitive and resistant *C. albicans*, the MICs of the KBN are consistently 6.25 mg/mL. This indicates that KBN has the same antifungal activity against both sensitive and resistant *C. albicans*.

### 2.3 Inhibitory effect of KBN combined with MN on *C. albicans*

The MIC values of KBN for *C. albicans* strains *901*, *904*, *953*, and *311* are 6.25 mg/mL, and the MIC values for MN against these strains are 2 µg/mL. When KBN concentrate and MN are used in combination against the drug-resistant strains *901*, *904*, *953*, and *311*, the FICI values are all 0.2498, indicating a synergistic interaction (**Table 4**). This suggests that the combination of KBN and MN is effective in restoring the sensitivity of drug-resistant strains. The experiment found that a low dose of KBN could restore the sensitivity of drug-resistant strains to MN, with significant and stable synergistic effects observed even at concentrations below 0.39 mg/mL (**Table 4**).

**Table 4:** The MIC values and FICI values of KBN and MN against *C. albicans*.

*C. albicans* Strains	KBN(mg/mL)	MN (µg/ml)	combined use		FICI
			KBN	MN	
**901**	6.25	2	0.78	0.25	0.2498
**904**	6.25	2	0.78	0.25	0.2498
**953**	6.25	2	0.78	0.25	0.2498
**311**	6.25	2	0.78	0.25	0.2498

*When FICI ≤ 0.5, it indicates a synergistic effect; when 0.5 < FICI ≤ 1, it indicates an additive effect; when 1 < FICI ≤ 4, it indicates no interaction; and when FICI > 4, it indicates an antagonistic effect.

The evaluation method for drug combination effects employs the Fractional Inhibitory Concentration Index (FICI) model. This model is based on the Loewe Additivity (LA) theory. The LA theory is predicated on the assumption that a drug does not interact with itself and requires that equivalent therapeutic effects are achieved for comparison purposes. Specifically, the FICI model compares the concentration of drugs used in combination to those used individually, as follows:


$$\begin{array}{c} \text{FICI}={\text{FICI}}_{\text{A}}+{\text{FICI}}_{\text{B}}\\ =\left({\text{MIC A}}_{\text{comb}}\right)/\left({\text{MIC A}}_{\text{alone}}\right)+\left({\text{MIC B}}_{\text{comb}}\right)/\left({\text{MIC B}}_{\text{alone}}\right) \end{array}$$

When FICI ≤ 0.5, it indicates a synergistic effect; when 0.5 < FICI ≤ 1, it indicates an additive effect; when 1 < FICI ≤ 4, it indicates no interaction; and when FICI > 4, it indicates an antagonistic effect.

### 2.4 The XTT assay evaluates the inhibitory effect on biofilms of the combination of KBN and MN

**Figure 4:**
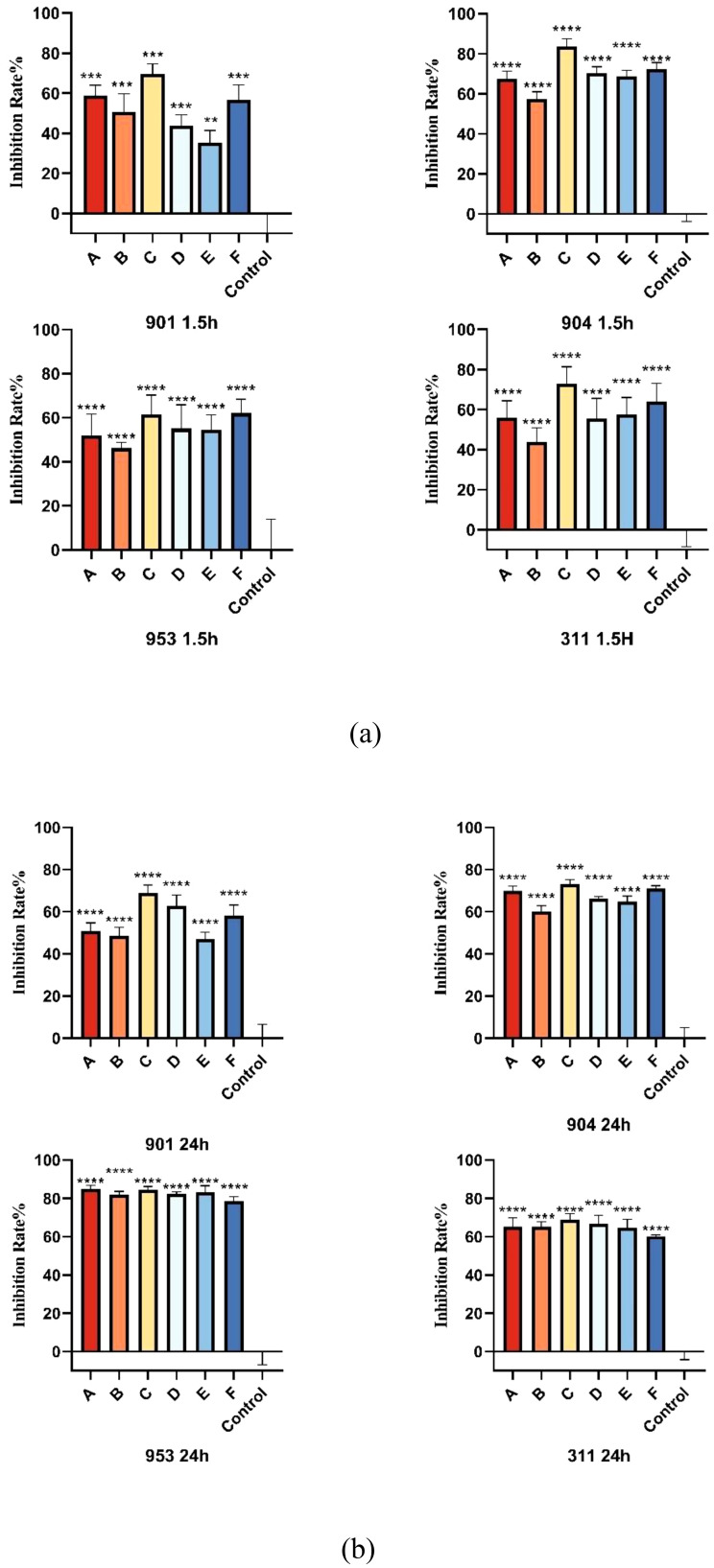
**(A)** The inhibition rates of early biofilms (1.5h) of *C. albicans 901*, *904*, *953*, and *311*; **(B)** The inhibition rates of mature biofilms (24h) of *C. albicans 901*, *904*, *953*, and *311*. A: KBN at 6.25 mg/mL, B: KBN at 0.78 mg/mL + MN at 0.25 µg/mL, C: KBN at 0.39 mg/mL + MN at 0.25 µg/mL, D: KBN at 0.19 mg/mL + MN at 0.25 µg/mL, and E: MN at 2 µg/mL. ** P ≤0.01; *** P ≤0.001; **** P ≤0.0001.

The XTT reduction assay is used to quantitatively determine the effect of the combination of KBN and MN on the biofilms and planktonic cells of *C. albicans*. The inhibitory effect on biofilms is calculated as a percentage of the metabolic activity compared to the blank control group. Three combined concentrations, as well as the MIC concentrations of KBN and MN, significantly reduce the metabolic activity of *C. albicans*. The concentration of KBN at 0.78 mg/mL combined with MN at 0.25 µg/mL demonstrates a significant and consistent inhibitory effect on both early and mature biofilms (**Figure 4**, **Tables 5**, **6**).

**Table 5:** The impact of KBN lotion in combination with MN on early biofilms (1.5 hours).

Groups Biofilm Inhibition %	901	904	953	311
**KBN 12.5mg/mL**	58.88 5.14	67.42 3.87	65.25 4.51	81.97 1.70
**KBN 6.25mg/mL**	50.70 8.93	57.30 3.73	65.25 2.52	84.86 1.92
**KBN 0.78mg/mL+ MN 0.25μg/mL**	56.78 7.39	68.66 3.09	64.77 4.32	83.10 3.50
**KBN 0.39mg/mL+ MN 0.25μg/mL**	43.69 5.50	70.16 3.26	66.58 4.53	82.25 1.34
**KBN 0.19mg/mL+ MN 0.25μg/mL**	35.28 6.11	72.16 3.40	68.87 3.08	84.30 1.93
**MN 2μg/mL**	69.63 4.98	83.52 3.85	60.17 0.72	78.66 2.18

(¯x ± s,n=6).

**Table 6:** The impact of KBN lotion in combination with MN on mature biofilms (24 hours).

Groups Biofilm Inhibition %	901	904	953	311
**KBN 12.5mg/mL**	58.88 5.14	67.42 3.87	65.25 4.51	81.97 1.70
**KBN 6.25mg/mL**	50.70 8.93	57.30 3.73	65.25 2.52	84.86 1.92

(¯x ± s,n=6).

### 2.5 Evaluation of the impact of combined use of KBN lotion and MN on *C. albicans* biofilms at early and mature stages by ROS assay

**Figure 5:**
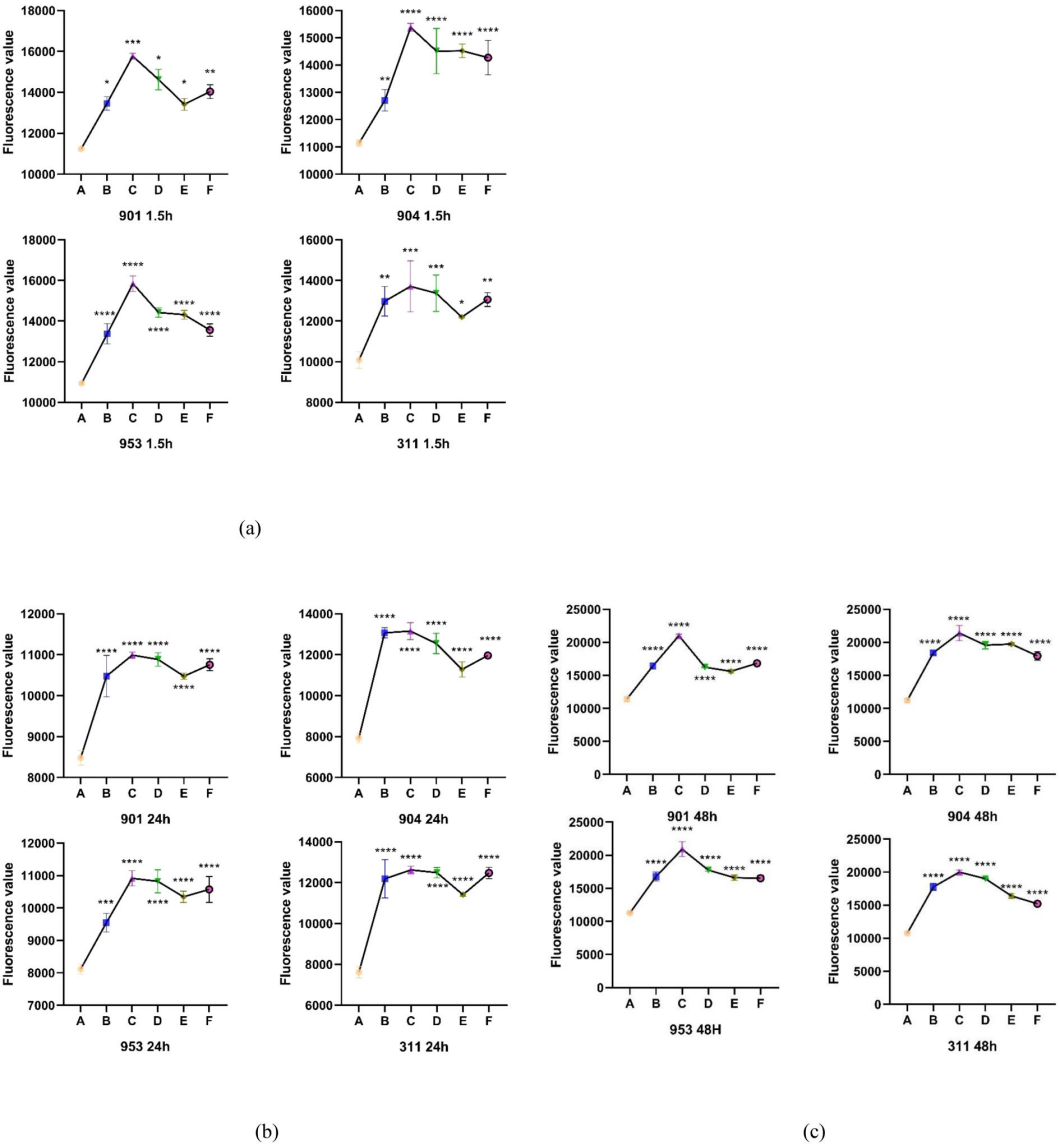
**(A)** The Fluorescence value of early biofilms (1.5h) of *C. albicans 901*, *904*, *953*, and *311*; **(B)** The Fluorescence value of mature biofilms (24h) of *C. albicans 901*, *904*, *953*, and *311*. **(C)** The Fluorescence value of mature biofilms(48h) of *C. albicans 901*, *904*, *953*, and *311*. A: KBN at 6.25 mg/mL, B: KBN at 0.78 mg/mL + MN at 0.25 µg/mL, C: KBN at 0.39 mg/mL + MN at 0.25 µg/mL, D: KBN at 0.19 mg/mL + MN at 0.25 µg/mL, and E MN at 2 µg/mL. * P ≤0.05; ** P ≤0.01; *** P ≤0.001; **** P ≤0.0001.

Utilizing DCFH-DA as a probe, the study assessed the accumulation of reactive oxygen species (ROS) in *C. albicans* biofilms at both early and mature stages across varying treatment concentrations. Observations revealed that for both initial and mature biofilm stages, fluorescence intensity significantly increased across all five treatment concentrations compared to the control group, indicating marked intracellular ROS production and accumulation. The combination therapy of KBN and MN, across high, medium, and low dosage tiers, matched the fluorescence intensities observed at the MIC levels of KBN and MN when used independently. Notably, the combined dosage of KBN at 0.78 mg/mL and MN at 0.25 µg/mL elicited the most pronounced increase in fluorescence intensity for both biofilm stages. This indicates that this specific concentration of combined therapy significantly enhanced ROS accumulation, inducing oxidative stress and effectively promoting fungal cell death (**Figure 5**, **Tables 7**–**9**)

**Table 7:** Fluorescence values of KBN in combination with MN on early biofilms (1.5 hours).

Groups Fluorescence value	901	904	953	311
**Control**	11233.67± 46.36	11141.00 117	10939.00 67.45	10084.67 409.06
**KBN 6.25mg/mL**	13457.00 325.12	12709.00 392.53	13378.33 500.31	12975.67 727.93
**KBN 0.78mg/mL+ MN 0.25µg/mL**	15101.67 1251.76	15384.67 150.28	15841.33 380.42	13713.00 1256.81
**KBN 0.39mg/mL+ MN 0.25µg/mL**	14631.33 505.38	14513.33 831.44	14421.67 233.29	13374.67 895.04
**KBN 0.19mg/mL+ MN 0.25µg/mL**	13946.67 672.07	14523.33 247.25	14316.33 216.77	12193.33 45.06
**MN 2µg/mL**	14036.33 333.98	14272.33 629.21	13561.00 303.79	13063.00 342.52

(¯x ± s,n=3).

**Table 8:** Fluorescence values of KBN in combination with MN on mature biofilms (24 hours).

Groups Fluorescence value	901	904	953	311
**Control**	8479.67± 177.86	7922.33± 231.53	8113.33± 156.59	7616.00± 275.80
**KBN 6.25mg/mL**	10478.00± 505.37	13070.67± 244.11	9545.67± 287.03	12191.33± 938.05
**KBN 0.78mg/mL+ MN 0.25µg/mL**	10991.00± 73.30	13157.000± 413.49	10916.67± 235.09	12621.00± 185.43
**KBN 0.39mg/mL+ MN 0.25µg/mL**	10884.33± 165.19	12551.33± 495.70	10824.33± 353.74	12491.33± 254.69
**KBN 0.19mg/mL+ MN 0.25µg/mL**	10475.33± 75.57	11283.33± 369.27	10345.00± 173.25	11413.00± 76.30
**MN 2µg/mL**	10756.67± 145.50	11965.67± 94.32	10572.67± 398.01	12476.33± 280.39

(¯x ± s,n=3).

**Table 9:** Fluorescence values of KBN in combination with MN on mature biofilms (48 hours).

Groups Fluorescence value	901	904	953	311
**Control**	11399.33±398.31	11220.33±299.37	11287.67±141.08	10767.00±83.72
**KBN 6.25mg/mL**	16418.00±473.34	18428.00±300.65	16788.67±673.47	17758.67±528.51
**KBN 0.78mg/mL+ MN 0.25µg/mL**	21015.33±218.22	21399.33±1162.9	20919.00±1126.09	20015.33±304.67
**KBN 0.39mg/mL+ MN 0.25µg/mL**	16246.33±90.94	19570.67±553.02	17756.00±180.03	19014.67±66.29
**KBN 0.19mg/mL+ MN 0.25µg/mL**	15615.67±275.95	19731.33±224.26	16616.33±421.92	16408.67±363.15
**MN 2µg/mL**	16823.33±362.15	17947.00±646.31	16540.33±392.27	15225.33±157.98

(¯x ± s,n=3).

### 2.6 Detecting the effect of combined use of KBN and MN on early and mature *C. albicans* biofilms by fluorescence microscopy

In this study, we used FITC-conA fluorescent dye to label the extracellular polysaccharides in *Candida albicans* biofilms, allowing us to observe the effects of drug treatment on their biofilm structure. FITC-conA binds to the extracellular polysaccharides in the *C. albicans* biofilm and exhibits a bright green fluorescence under a fluorescence microscope, enabling a clear visual assessment of the biofilm’s thickness and structural integrity. As the culture time increases, the biofilm in the control group progressively thickens, forming clumps characterized by buds encased in secretions and a network of intertwined fungal hyphae, resulting in dense, flaky biofilms and hyphal structures visible under the microscope. However, the combined application of KBN and MN significantly disrupts the biofilm structure of *C. albicans*, primarily revealing scattered spores of the fungus. These spores exhibit irregular morphology and size variation, with only a few inhibited hyphae visible and no dense biofilm formations observed.

**Figure 6:**
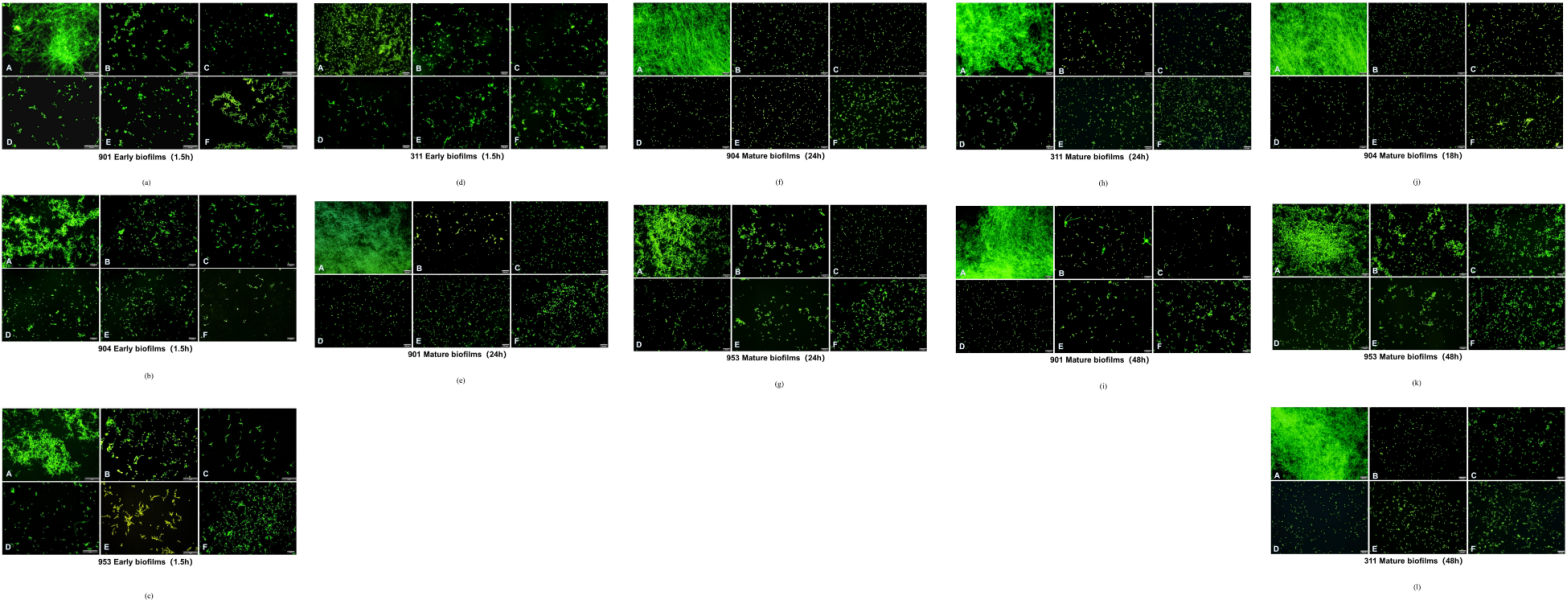
Fluorescence microscopy images of *C. albicans* after early stage (1.5h) **(A–D)** and mature stages [24h **(E–H)**, 48h **(I–L)**] treatment with A: blank RMPI 1640 liquid medium, B: KBN at 6.25 mg/mL, C: KBN at 0.78 mg/mL + MN at 0.25 µg/mL, D: KBN at 0.39 mg/mL + MN at 0.25 µg/mL, E: KBN at 0.19 mg/mL + MN at 0.25 µg/mL, and F: MN at 2 µg/mL. The scale bar represents 50 micrometers.

Across all stages of biofilm development—early, mature, and late mature—the combined use of KBN and MN at varying doses achieves the effectiveness of the MIC levels seen with individual applications of KBN and MN. Notably, the combination of KBN at 0.78 mg/mL and MN at 0.25 µg/mL demonstrates superior efficacy in disrupting the biofilm structure of *C. albicans* compared to the individual MIC levels of either agent. This tailored approach not only showcases the enhanced disruption of fungal biofilms but also highlights the potential for more effective antifungal strategies through combined therapeutic dosing (**Figure 6**).

### 2.7 Detecting the effect of combined use of KBN and MN on the viability of *C. albicans* within early and mature biofilms by CLSM

Confocal laser scanning microscopy (CLSM) has further validated the antimicrobial efficacy of the KBN and MN combination. In this study, we employed the SYTO 9/PI Live/Dead Bacterial Double Staining Kit for cell viability assessment. Propidium iodide (PI), which can only infiltrate cells with compromised membranes, marks dead cells with a red fluorescence. In contrast, live cells, characterized by intact membranes, emit a bright green fluorescence. A greater proportion of damaged fungi results in a more pronounced orange hue due to the overlay of these fluorescent signals.

**Figure 7:**
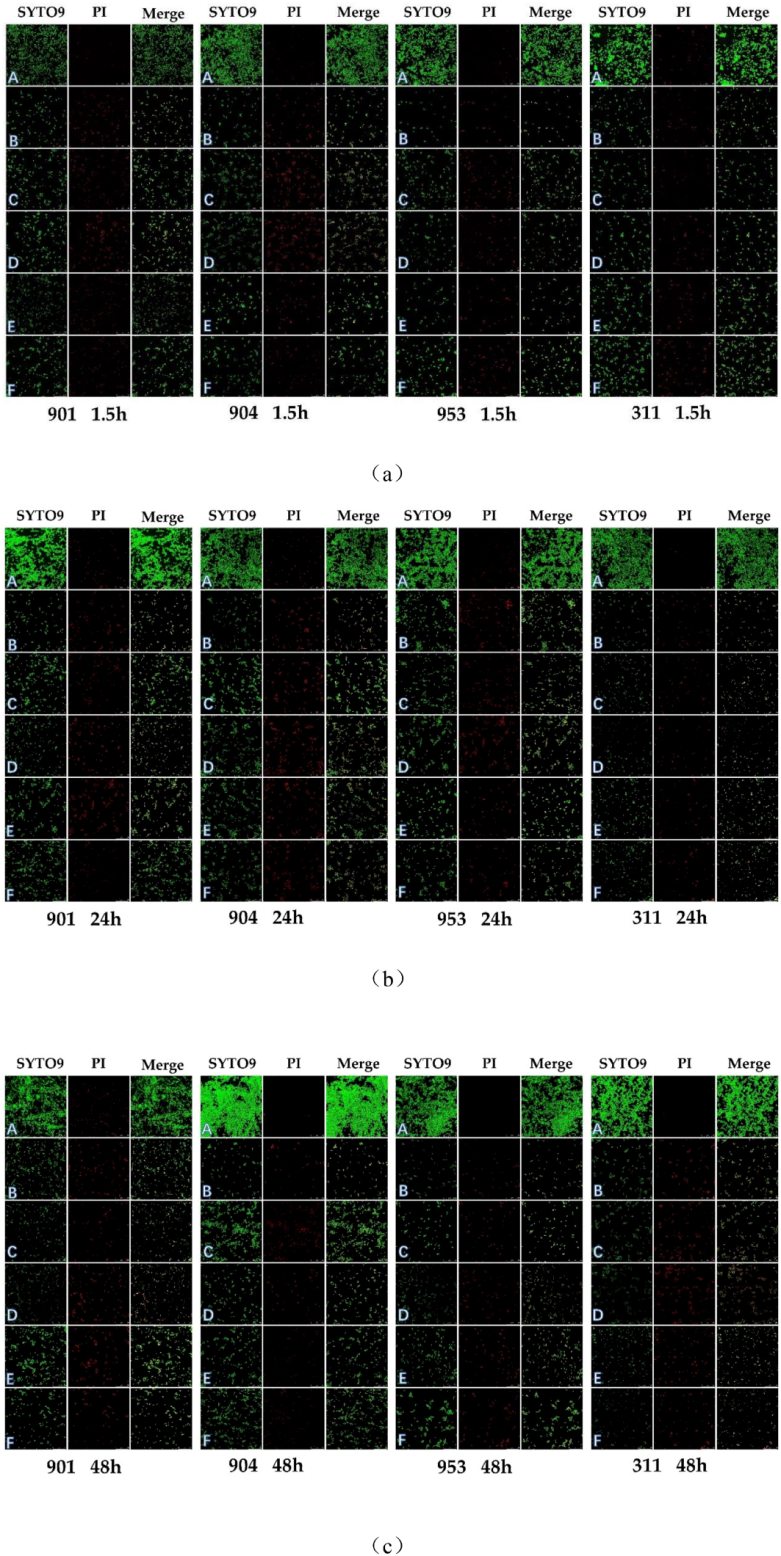
CLSM images of (*C. albicans* after early (1.5h) **(A)** and mature stages [24h **(B)**, 48h **(C)**] treatment with A: blank RMPI 1640 liquid medium, B: KBN at 6.25 mg/mL, C: KBN at 0.78 mg/mL + MN at 0.25 µg/mL, D: KBN at 0.39 mg/mL + MN at 0.25 µg/mL, E: KBN at 0.19 mg/mL + MN at 0.25 µg/mL, and F: MN at 2 µg/mL. SYTO 9 emits green fluorescence indicating live cells within intact biofilms; PI emits red fluorescence indicating dead cells within damaged biofilms; the combined orange fluorescence intensity represents the proportion of live to dead cells. The scale bar represents 50 micrometers.

In the untreated control group, early and mature stages of *C. albicans* biofilms were observed under the microscope as dense clusters emitting bright green fluorescence, with negligible red fluorescence. For the samples treated solely with KBN detergent, solely with MN, and with a combination of KBN and MN at three different dosages, the *C. albicans* biofilms predominantly consisted of dispersed yeast cells and pseudohyphae, with an almost complete absence of true hyphae and no dense structural formations. There was a noticeable increase in red fluorescence, indicating cell damage, alongside a significant decrease in green fluorescence, indicating fewer live cells. The combination treatment of KBN at 0.78 mg/mL and MN at 0.25 µg/mL showed the highest proportion of red fluorescence, suggesting the most effective biofilm disruption (**Figure 7**).

**Figure 8:**
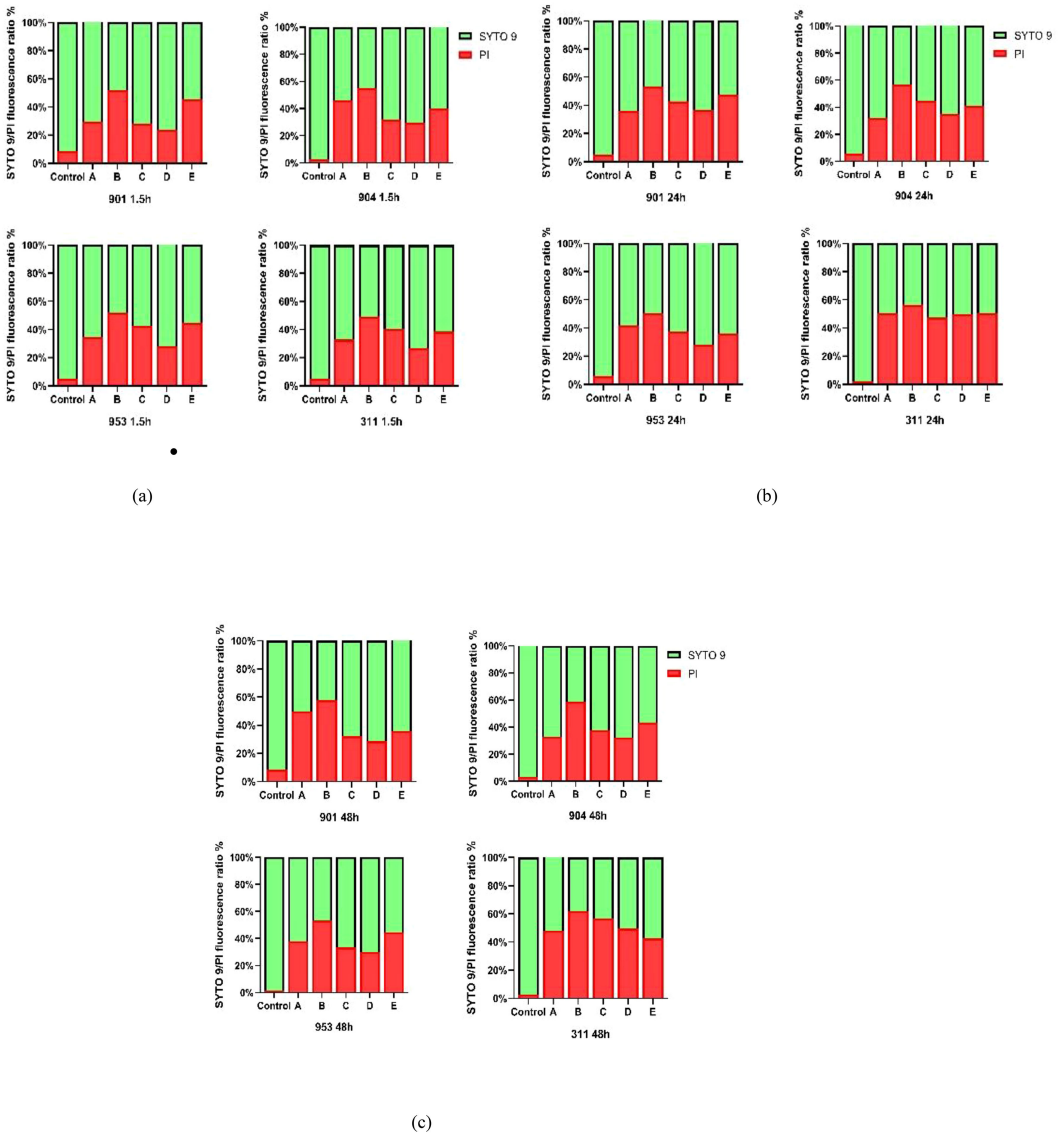
**(A)** The SYTO 9/PI fluorescence ratio of early biofilms (1.5h) of (*C. albicans 901, 904, 953*, and *311*; **(B)** The SYTO 9/PI fluorescence ratio of mature biofilms (24h) of *C. albicans 901, 904, 953*, and *311*.**(C)** The SYTO 9/PI fluorescence ratio of mature biofilms (48h) of (*C. albicans 901, 904, 953*, and *311*. A: KBN at 6.25 mg/mL, B: KBN at 0.78 mg/mL + MN at 0.25 µg/mL, C: KBN at 0.39 mg/mL + MN at 0.25 µg/mL, D: KBN at 0.19 mg/mL + MN at 0.25 µg/mL, and E: MN at 2 µg/mL.

Utilizing Image J software for fluorescence intensity quantification from confocal laser scanning microscope images, the ratio of green to red fluorescence provided an accurate measure of biofilm alterations post-drug treatment. In the control group, the SYTO 9/PI fluorescence ratio demonstrated high fungal vitality within resistant *C. albicans* biofilms across early and mature stages, underlining minimal cell mortality protected by biofilm. In contrast, drug-treated groups exhibited a notable decline in green fluorescence alongside an uptick in red fluorescence, indicating a substantial increase in cell mortality and a decrease in viable cell count. Particularly, the drug combination of KBN 0.78mg/mL and MN 0.25µg/mL showcased the highest red fluorescence ratio, implying superior efficacy in biofilm disruption and fungal cell death. This effect was consistent across all biofilm stages, with early biofilm red fluorescence ratios for strains 901, 904, 953, and 311 at 51.75%, 55.06%, 51.90%, and 49.29% respectively; at the mature stage (24h), ratios were 53.53%, 57.13%, 50.56%, and 56.66% respectively; and at the mature stage (48h), they reached 57.85%, 58.68%, 53.24%, and 61.92% respectively. These findings underscore the combination therapy’s potent impact on biofilm structural integrity and fungal mortality, highlighting its synergistic effect against biofilms (**Figure 8**).

### 2.8 Expression analysis of KBN and MN on *ALS1*, *ALS3*, *HWP1*, *HSP90* and *CSH1* of *C.albicans* biofilm formation by RT-PCR

**Figure 9:**
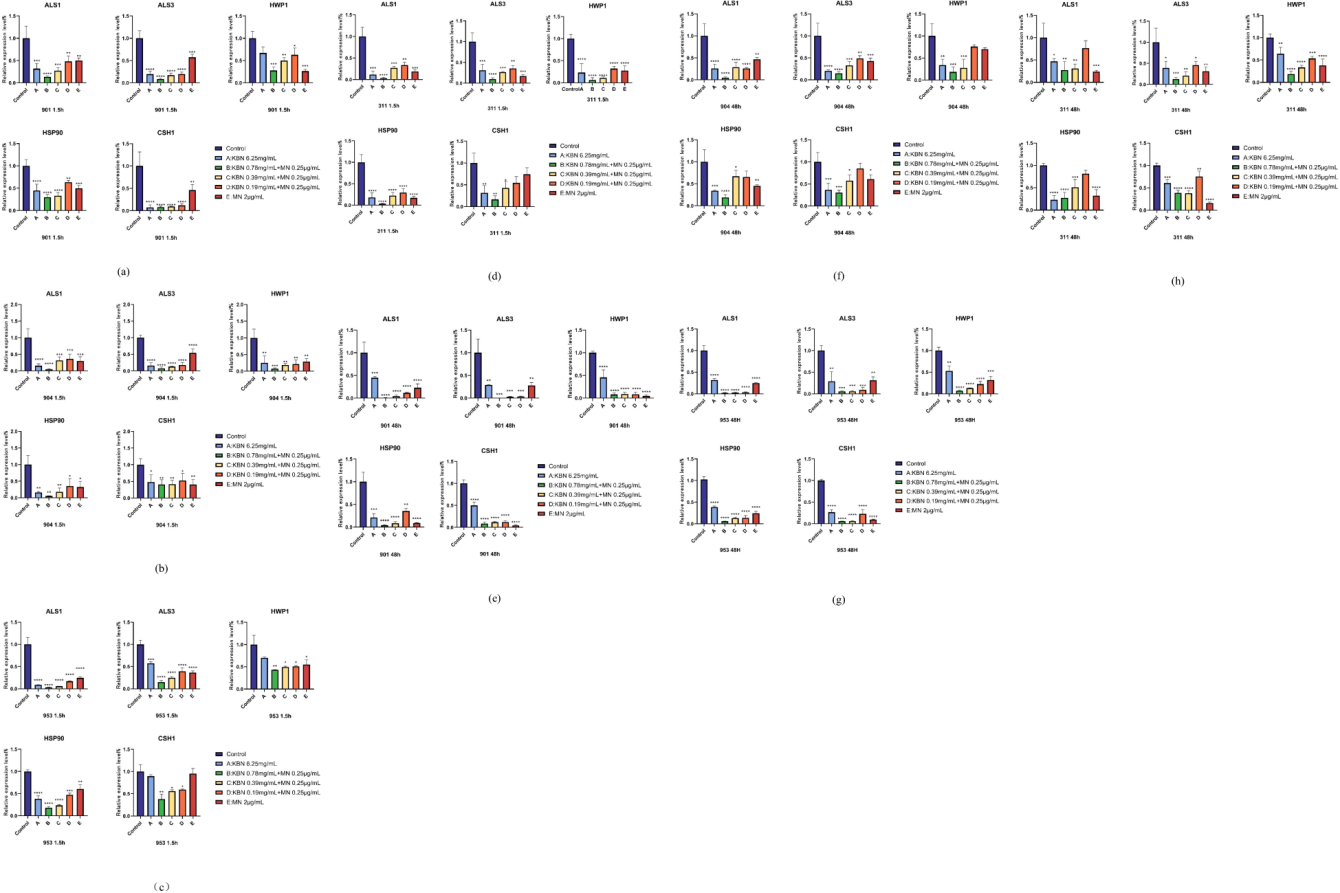
Comparison of the relative expression levels of the *ALS1*, *ALS3*, *HWP1*, *Hsp90*, *CSH1*gene, associated with the formation of *C. albicans* biofilms, after early (1.5 hours) **(A–D)** and mature (48 hours) **(E–H)** stages treatment with KBN at 6.25 mg/mL, KBN 0.78 mg/mL + MN 0.25 µg/mL, KBN 0.39 mg/mL + MN 0.25 µg/mL, KBN 0.19 mg/mL + MN 0.25 µg/mL, MN at 2 µg/mL, and a control of blank 1640 liquid medium. Expression of the individual genes was normalized to the expression of *18s*, a reference gene, and is shown relative to untreated control cells. * P ≤0.05; ** P ≤0.01; *** P ≤0.001; **** P < 0.0001.

Given the observed inhibitory impact of KBN and MN on *C. albicans* biofilms, we delved into the expression patterns of several key genes known to play roles in hyphal development and adherence. The findings from RT-PCR revealed that post-treatment, there was a notable decrease in the expression levels of *ALS1*, *ALS3*, *HWP1*, *HSP90*, and *CSH1*. The efficacy observed at the three combined concentrations matched that of the MIC values when KBN and MN were used individually. Notably, the combined dose of KBN at 0.78 mg/mL and MN at 0.25 µg/mL exhibited the most pronounced effect in terms of gene suppression, surpassing the downregulation observed with the individual MIC doses of either KBN or MN (**Figure 9**).

## 4 Discussion

*Candida albicans*, typically commensal microorganisms colonizing the skin, vaginal, gastrointestinal, and pharyngeal cavities, can transition to opportunistic pathogens under certain conditions, leading to infections ranging from superficial to life-threatening systemic candidiasis. The virulence of these infections is significantly enhanced by the formation of biofilms, which protect the fungal cells from antifungal agents and immune responses, contributing to high mortality rates, particularly in immunocompromised patients​ (Atiencia-Carrera et al., 2022b; Yu et al., 2022)​. Biofilms increase the resistance of *Candida albicans* to first-line antifungals, complicating treatment efforts​ (Zhu et al., 2022).

In tackling antifungal resistance, especially in the context of *C. albicans* biofilms, our study adds to the essential discussion on the effectiveness of combination therapies. The synergistic interaction between KBN and MN observed in our research is consistent with recent findings advocating for combination therapies to improve antifungal efficacy​ (Atiencia-Carrera et al., 2022a; Atiencia-Carrera et al., 2022b). This strategy is not only a current focal point but also represents a significant trend in future therapeutic developments (Liu et al., 2014; Czechowicz et al., 2021)​.

Alternative treatments targeting biofilm formation and maintenance are crucial. These approaches, including new antifungal agents and combination therapies (Wang et al., 2021), are essential for reducing morbidity and mortality associated with biofilm-related infections (Guo et al., 2008). The demonstrated effectiveness of KBN and MN combination therapy in our study underscores the potential of these approaches to surpass the limitations of existing treatments and offer more robust solutions for combating *Candida* infections​.

The KBN lotion is composed of Huanglian, Sanbaicao, Daqingye, Jiguanghua, Xiangru, Kushen, Baibu, Longdan, Dingxiang, and Bingpian. It possesses the efficacy of clearing heat, drying dampness, and killing insects to alleviate itching. Huanglian in the prescription contains berberine, palmatine, berberine alkaloid, and epiberberine. Its bacteriostatic main components are berberine and berberine alkaloid. Among them, berberine can inhibit the formation of *C. albicans* biofilms by downregulating the expression levels of *EFG1*, *HWP1*, *ALS1*, and *ECE1* genes. *EFG1* regulates the transition to hyphal growth, essential for biofilm development. *HWP1* and *ALS1* are involved in adhesion, crucial for biofilm stability, while *ECE1* contributes to biofilm maturation and immune evasion. By suppressing these genes, berberine disrupts biofilm formation, reducing the pathogen’s virulence and resistance to antifungal treatments. This inhibitory effect of berberine hydrochloride on *C. albicans* biofilm formation was demonstrated in the study by Huang et al. (Huang et al., 2020). The main active ingredient in Sanbaicao is Sauchinone, which exhibits anti-inflammatory effects by inhibiting the activity of NF-κB and reducing TNF-α expression in macrophages (Hwang et al., 2003). Daqingye’s effective antibacterial component is indirubin, which can effectively inhibit the formation of Candida albicans mixed biofilms and significantly suppress the activity of *C.albicans* mixed biofilm formation, demonstrating good efficacy in the prevention and treatment of VVC (Ahmad et al., 2010; Ponnusamy et al., 2010). The main component of Jiguanghua, kaempferol, has strong anti-inflammatory effects, regulating the activity of pro-inflammatory enzymes and the expression of inflammation-related genes (Devi et al., 2015). The primary antibacterial active ingredient in Dingxiang is eugenol, which can disrupt the fungal cell membrane (He et al. 2007; Zanul Adibin et al. 2023).

The noteworthy reduction in MIC values achieved through the concurrent application of KBN and MN underscores a potent synergistic effect, resonating with the work of Czechowicz, P et al. (Kim and Lee, 2021), who documented enhanced biofilm disruption with low-dose combination treatments. This approach highlights the potential of sub-inhibitory concentrations to target and dismantle biofilm-associated resistance mechanisms, thereby restoring fungal susceptibility to antifungal agents.

Furthermore, several studies have highlighted the significant role of oxidative stress in the antifungal action against Candida albicans, particularly within biofilms. Yanjiao Ding et al. (Hwang et al., 2012) demonstrated that D319 induces ROS-mediated apoptosis by inhibiting isocitrate lyase, leading to mitochondrial dysfunction and cell death. Ji Hong Hwang et al. (Tian et al., 2019)showed that (+)-Medioresinol promotes ROS accumulation, triggering mitochondria-mediated apoptosis. Similarly, Heesu K et al. (Ding et al., 2022) emphasized the role of nanoparticles in inducing oxidative stress, disrupting fungal cell wall integrity, and promoting cell death through ROS production. These findings support our observations of the KBN and MN combination’s efficacy, reinforcing oxidative stress as a critical mechanism for reducing fungal viability and enhancing treatment outcomes.

In this study, the use of FITC-conA (Galdiero et al., 2021) for the visualization of *C. albicans* biofilm disruption through the combined application of KBN and MN dovetails with the growing body of research highlighting the susceptibility of biofilms to antifungal agents. Our findings underscore the effectiveness of synergistic antifungal combinations in compromising biofilm integrity, particularly evident in the significant disruption caused by the KBN 0.78 mg/mL and MN 0.25 µg/mL dosage. This synergy is further validated by our confocal CLSM analysis, which, akin to the approach by Lee and Park (Li et al., 2019; Ma et al., 2020), utilizes SYTO 9/PI staining to differentiate between viable and compromised fungal cells within biofilms. The notable increase in red fluorescence in treated samples—indicative of enhanced cellular damage—positions this combination therapy as a viable antifungal strategy, supporting the conclusions drawn by Patel and Wright (Gonçalves et al., 2015; Qian et al., 2020; Bonvicini et al., 2021) in their review of fungal biofilm resistance mechanisms. This alignment with contemporary research (Hoyer et al., 1998) underscores the potential of our findings to contribute to the development of more effective treatments against fungal biofilms, with the caveat that further studies are needed to explore the broader implications of these synergistic treatments across various fungal pathogens.

In the context of our findings on the inhibitory effects of KBN combined with MN on *C. albicans* biofilms, we further explored the expression of genes implicated in hyphal formation and adhesion. The results from RT-PCR revealed a significant downregulation of *ALS1*, *ALS3*, *HWP1*, *HSP90*, and *CSH1* following treatment. The efficacy observed at the three combined concentrations matched that of the MIC values when KBN and MN were used individually, with the combination of KBN at 0.78 mg/mL and MN at 0.25 µg/mL showing the most pronounced effect on gene suppression. This downregulation surpasses the effects seen with individual applications of KBN and MN at their respective MIC concentrations.

The significant downregulation of *ALS1*, *ALS3*, *HWP1*, *HSP90*, and *CSH1* post-treatment aligns with the hypothesis that targeting specific pathways related to biofilm integrity and fungal virulence can enhance the therapeutic efficacy against *C. albicans*. The *ALS* gene family, particularly *ALS1* and *ALS3* (Liu and Filler, 2011; Roudbarmohammadi et al., 2016), plays a crucial role in the adhesion process, essential for biofilm formation and maintenance (Murad et al., 2001). *HWP1* is integral to hyphal development, another critical component of biofilm structure (Murzyn et al., 2010a; Oh et al., 2010b; Salehipour et al., 2021). *Hsp90*, a heat shock protein, is known to stabilize several host proteins and has been implicated in stress response pathways that contribute to anti-fungal resistance (Yan et al., 2019; Huang et al., 2020). Murzyn, A, Yan, Y, et al. have also demonstrated a positive correlation between *CSH1* expression and biofilm formation (Wall et al., 2019; Lu et al., 2021). The observed downregulation of these genes suggests that the combination therapy not only disrupts biofilm formation but also impacts the fungal cell’s ability to adhere and form hyphae, key factors in its pathogenicity and resistance.

Our study provides insights into the potential molecular mechanisms behind *C. albicans* ‘ resistance, suggesting that combination therapy, particularly through gene expression modulation, could offer a promising approach to address fungal infections. The observed synergistic effect in downregulating genes essential for biofilm formation and virility, achieved with KBN and MN, points towards a strategic avenue that might help in navigating the complexities of antifungal resistance. This prompts a call for further explorations into the specific molecular interactions spurred by this therapy, with the goal of uncovering new therapeutic targets (Romo et al., 2017; Wall et al., 2019) that could enhance antifungal strategies. Aligning with the evolving landscape of current re-search (Pohl, 2022), there appears to be a consensus on the potential benefits of pursuing combination therapies against fungal biofilms. Recent discussions in the field (Chen et al., 2020), as outlined by reviews, emphasize a nuanced understanding of biofilm dynamics and the identification of molecular targets as essential steps towards crafting more nuanced antifungal treatments (Xu et al., 2014). Our research adds to the dialogue on addressing the challenge of biofilm-mediated antifungal resistance, highlighting the prospective value of innovative therapeutic approaches in the broader context of combating fungal infections.

## 5 Conclusions

To address the challenge of antifungal resistance, especially against *C. albicans* biofilms, our study conducted *in vitro* experiments on the combined use of KBN and MN against *C. albicans* biofilms, demonstrating a synergistic effect in combating these biofilms. Through detailed analysis using FITC-conA for biofilm visualization and real-time RT-PCR for gene expression, our findings indicate that this combination therapy not only disrupts the integrity of the biofilms but also significantly downregulates key genes related to fungal adhesion and hyphal formation. These observations suggest that targeting specific fungal pathways and mechanisms through combined therapeutic strategies can significantly improve the management of fungal infections, offering a promising route to overcome the complexities of antifungal resistance. This also provides experimental support for future clinical applications of KBN in combination with antibiotics to treat infections caused by drug-resistant *C. albicans*. Despite the promising *in vitro* results of our study on the synergistic effect of KBN and MN against *C. albicans* biofilms, our experiment did not validate these effects in drug-resistant *C. albicans* VVC cell and animal models. Furthermore, the study did not investigate the specific mechanisms behind the synergy between KBN and MN or the interactions that might impact efficacy and safety. Future research should address these limitations to confirm the findings and understand the therapeutic potential of this combination.

## Data Availability

The original contributions presented in the study are included in the article/**Supplementary Material**. Further inquiries can be directed to the corresponding authors.
