# Supplementary material for: Synergistic in vitro activity and mechanism of KBN lotion and miconazole nitrate against drug-resistant Candida albicans biofilms
Source: Front Cell Infect Microbiol. 2024 Aug 29;14:1426791. doi: 10.3389/fcimb.2024.1426791 (PMC11390680; doi:10.3389/fcimb.2024.1426791)
Supplement: Supplementary file 1 [file Table1.docx]

# Resubmission Statement: Revision Instructions for Characterizing the Components and Active Substances in KBN Lotion

To address the previous concerns regarding the unclear composition and active substances in KBN lotion, we have conducted detailed separation and characterization experiments as outlined below:

1. Sample Preparation:

Weigh 0.50 g of KBN lotion and ultrasonically extract it in 10 ml of 50% methanol-water for 1 hour. Subsequently, filter out the residue. Centrifuge the extract at 4°C and 12,000 rpm for 15 minutes, then filter the supernatant through a 0.22 μm membrane. Dilute the filtrate 5 times before performing UPLC-Q-TOF/MS analysis.

2. UPLC-Q-TOF/MS Analysis:

The composition of the two mobile phases was 0.1% (v/v) formic acid in water (A) and acetonitrile (B):0–15 min, 5%–35% B; 15–20 min, 35%–90% B; 20–28 min, 90%–5% B; 28–34 min, 5% B.The separations were performed with a constant flow rate of 0.3 ml/min.

Scanning Mode: +ESI and -ESI

Acquisition Range: Mass 50-1500 M/Z

Capillary Voltage ISVF: 5500V

Cone Voltage CE: ±35V

Ion Source Temperature: 550°C

Cone Gas Flow: 50 L/H

Injection Volume: 1 µL

3. Results:

The active compounds in KBN lotion were identified using UPLC-Q-TOF/MS analysis, which also provided the total ion chromatograms (Figure 1A, B). A total of 44 compounds were identified, including 16 alkaloids, 13 flavonoids, 4 phenols, 2 essential oils, 2 caffeoylquinic acids, 1 organic acid, 1 lignan, 1 tannin, 1 nucleosides, and 1 sugar (Table 1). According to the results of UPLC/Q-TOF MS, the main components of KBN lotion are alkaloids and flavonoids, many of which exhibit antibacterial activity.


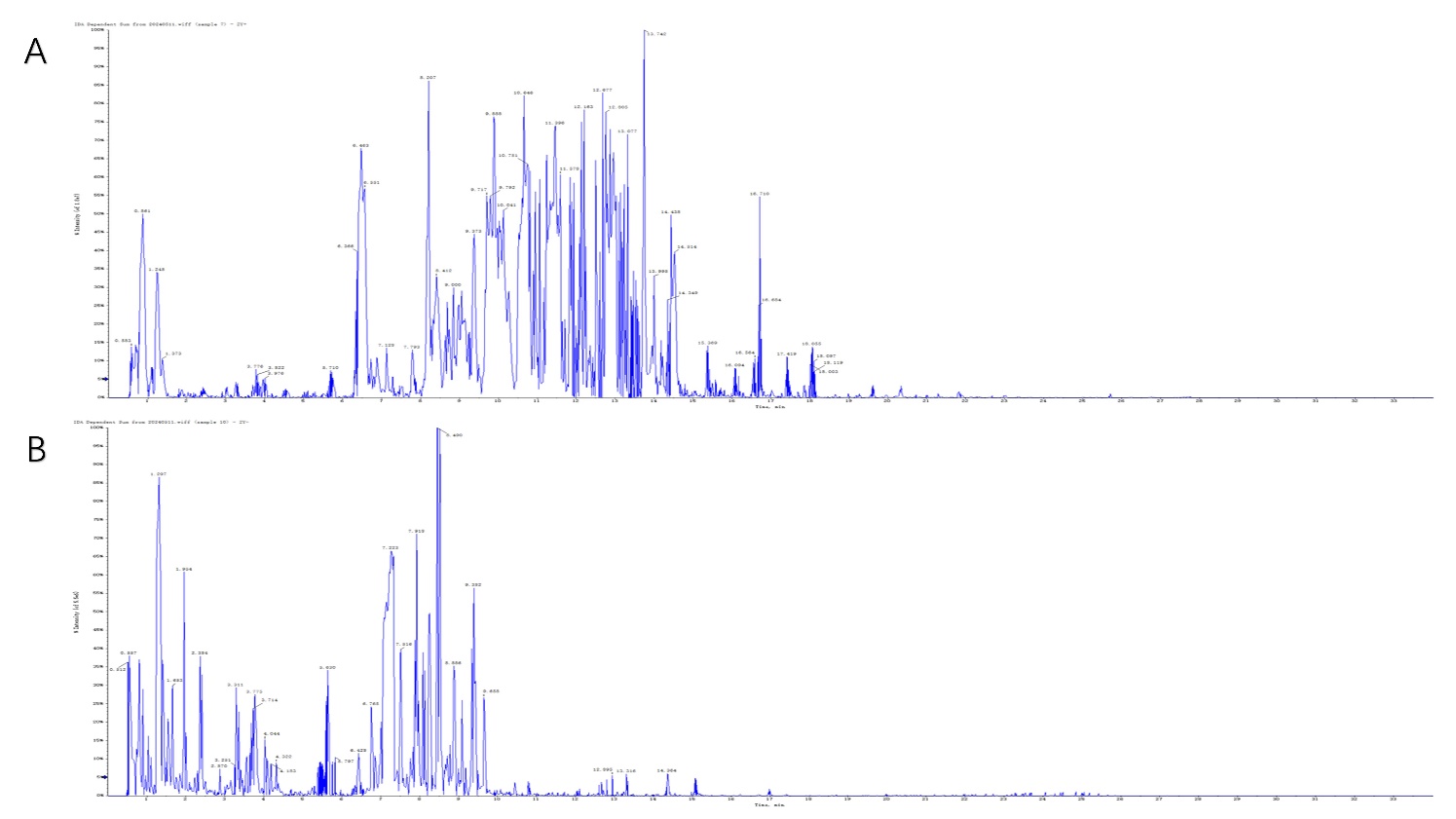


**Figure 1.** The total ion chromatogram obtained in the positive (A) and negative (B) modes.

**Table 1. Components of KBN lotion**

| **No** | **RT (min)** | **Extracting ions** | **Measured mass (m/z)** | **Formula** | **Identity** | **Error (ppm)** | **Main secondary fragment ions (MS/MS)** | **Category** | **Cas no** |
| --- | --- | --- | --- | --- | --- | --- | --- | --- | --- |
| 1 | 0.6241333 | [M-H]- | 341.108 | C12H22O11 | Sucrose | -2.8 | 179,89,119 | Sugar | 57-50-1 |
| 2 | 0.7882167 | [M+H]+ | 265.1921 | C15H24N2O2 | 13a-Hydroxylupanin | 3.8 | 148,150,247 | Alkaloids | 15358-48-2 |
| 3 | 0.9240333 | [M+H]+ | 247.1801 | C15H22N2O | Sophocarpine | -1.6 | 245,179,136 | Alkaloids | 6483-15-4 |
| 4 | 1.018617 | [M-H]- | 169.0095 | C7H6O5 | Gallic acid | -26.6 | 125 | Tannins | 149-91-7 |
| 5 | 1.132767 | [M-H]- | 133.011 | C4H6O5 | Malic acid | -22.6 | 115,71 | Organic acids | 6915-15-7 |
| 6 | 1.17335 | [M+H]+ | 249.1974 | C15H24N2O | Matrine | 5.2 | 148,150 | Alkaloids | 519-02-8 |
| 7 | 1.264683 | [M+H]+ | 265.1938 | C15H24N2O2 | Oxymatrine | 14.3 | 247,205,148,136 | Alkaloids | 16837-52-8 |
| 8 | 1.4005 | [M+H]+ | 268.1068 | C10H13N5O4 | Adenosine | 10.4 | 136,119 | Nucleosides | 58-61-7 |
| 9 | 1.430583 | [M-H]- | 151.0361 | C8H8O3 | 4-hydroxyphenylacetic acid | -20.5 | 123,107 | Phenols | 156-38-7 |
| 10 | 2.37285 | [M-H]- | 153.0151 | C7H6O4 | Protocatechuic acid | -27.6 | 109,108 | Phenols | 99-50-3 |
| 11c | 3.809183 | [M+H]+ | 377.1431 | C16H24O10 | loganic acid | 8.2 | 213,169 | Terpenes | 22255-40-9 |
| 12a | 4.73045/14.24/17.023 | [M+H]+ | 165.0929 | C10H12O2 | Isoeugenol | 11.5 | 91,77 | Essential oils | 97-54-1 |
| 13 | 5.308917 | [M+H]+ | 338.1981 | C18H27NO5 | platyphylline | -5.6 | 236,264 | Alkaloids | 480-78-4 |
| 14a | 5.707/6.88765 | [M+H]+ | 355.1013 | C16H18O9 | Chlorogenic Acid | -3.0 | 235,205,319 | Caffeoylquinic Acids | 327-97-9 |
| 15ac | 5.716383/6.89 | [M+H]+ | 355.1042 | C16H18O9 | chlorogenic acid | 3.2 | 235,205,259,319 | Caffeoylquinic Acids | 327-97-9 |
| 16 | 6.530334 | [M+H]+ | 342.1727 | C20H23NO4 | Isocorydine | 7.9 | 279,265,237 | Alkaloids | 475-67-2 |
| 17 | 6.858217 | [M-H]- | 137.0204 | C7H6O3 | Salicylic acid | -29.3 | 93 | Phenols | 69-72-7 |
| 18 | 7.454433 | [M+H]+ | 195.0656 | C10H10O4 | Vanillin acetate | 2.1 | 77,91,103 | Essential oils | 881-68-5 |
| 19 | 7.800583 | [M+Na]+ | 379.1007 | C16H20O9 | Gentiopicrin | 1.8 | 158,200,217 | Terpenes | 20831-76-9 |
| 20 | 7.91575 | [M+Na]+ | 349.1379 | C20H22O4 | Licarin A | -6.0 | 137,272,314 | Lignans | 51020-86-1 |
| 21 | 8.696016 | [M+H]+ | 625.1718 | C28H32O16 | Isorhamnetin 3-glucoside-7-rhamnoside | 2.9 | 343,445,463,313 | Flavonoids | 17331-71-4 |
| 22 | 8.90575 | [M-H]- | 359.0692 | C18H16O8 | Rosmarinic acid | -22.4 | 161,197,179 | Phenols | 20283-92-5 |
| 23 | 9.063 | [M+H]+ | 340.1571 | C20H21NO4 | Papaverine | 8.1 | 324,309 | Alkaloids | 58-74-2 |
| 24 | 9.154333 | [M+H]+ | 187.0883 | C11H10N2O | Deoxyvasicinone | 9.1 | 187,118,120 | Alkaloids | 530-53-0 |
| 25 | 9.40765 | [M+NH4]+ | 372.1823 | C21H22O5 | Xanthohumol | 4.7 | 222,189,161 | Flavonoids | 6754-58-1 |
| 26 | 9.545466 | [M+H]+ | 287.056 | C15H10O6 | Kaempferol | 3.5 | 153,165,213 | Flavonoids | 520-18-3 |
| 27 | 9.6158 | [M+H]+ | 285.0789 | C16H12O5 | Calycosin | -3.8 | 270,168,140 | Flavonoids | 20575-57-9 |
| 28 | 9.639133 | [M]+ | 337.1278 | C20H18NO4 | Berberine | -9.1 | 321,292,278 | Alkaloids | 2086-83-1 |
| 29 | 10.0696 | [M+H]+ | 324.1256 | C19H17NO4 | Stylopine | 7.9 | 309,294,266 | Alkaloids | 84-39-9 |
| 30a | 10.086/16.7185 | [M+H]+ | 352.1183 | C20H17NO5 | Oxoglaucine | 0.8 | 322,337,294,308 | Alkaloids | 5574-24-3 |
| 31a | 10.0921/16.707 | [M+H]+ | 352.1199 | C20H17NO5 | Oxyberberine | 5.5 | 336,308,322,294 | Alkaloids | 19716-60-0 |
| 32 | 10.15977 | [M+H]+ | 338.1374 | C20H19NO4 | Dihydroberberine | -3.8 | 322,307,306 | Alkaloids | 483-15-8 |
| 33 | 10.15977 | [M+Na]+ | 397.1128 | C16H22O10 | Swertiamarin | 7.1 | 301,235,205 | Alkaloids | 17388-39-5 |
| 34 | 10.18243 | [M+H]+ | 354.1724 | C21H23NO4 | Dihydropalmatine | 6.7 | 338,323 | Alkaloids | 26067-60-7 |
| 35 | 10.48223 | [M+H]+ | 303.0499 | C15H10O7 | Quercetin | -0.1 | 229,153 | Flavonoids | 117-39-5 |
| 36 | 10.90972 | [M+H]+ | 317.0668 | C16H12O7 | Isorhamnetin | 3.8 | 302,153,170 | Flavonoids | 480-19-3 |
| 37 | 14.0955 | [M+H]+ | 285.0705 | C16H12O5 | Maackiain | 1.7 | 270,168 | Flavonoids | 2035-15-6 |
| 38 | 14.68963 | [M+H]+ | 269.0817 | C16H12O4 | Formononetine | -1.4 | 197,253,213,237 | Flavonoids | 485-72-3 |
| 39 | 15.38308 | [M+H]+ | 285.0786 | C16H12O5 | Wogonin | 10.0 | 168,140 | Flavonoids | 632-85-9 |
| 40 | 16.5545 | [M+H]+ | 263.0821 | C16H10N2O2 | Indirubin | 0.4 | 235,219,206,132 | Alkaloids | 479-41-4 |
| 41 | 16.7185 | [M+H]+ | 455.2128 | C26H30O7 | Kushenol I | 6.2 | 179,303,153,285,313 | Flavonoids | 99119-69-4 |
| 42 | 17.023333 | [M+H]+ | 165.0913 | C10H12O2 | Eugenol | 1.8 | 109,124,137,81 | Flavonoids | 97-53-0 |
| 43 | 18.98185 | [M+H]+ | 453.225 | C27H32O6 | 2'-O-methyl-Kurarinone | 11.0 | 179,329,303 | Flavonoids | 270249-38-2 |
| 44 | 21.89415 | [M+H]+ | 439.209 | C26H30O6 | Kurarinone | -2.3 | 179,303 | Flavonoids | 34981-26-5 |

4. Main Components and Their Activities:

According to the results of UPLC/Q-TOF MS, the main components of KBN lotion are alkaloids and flavonoids, many of which exhibit antibacterial activity. The KBN lotion is composed of Huanglian, Sanbaicao, Daqingye, Jiguanghua, Xiangru, Kushen, Baibu, Longdan, Dingxiang, and Bingpian. It possesses the efficacy of clearing heat, drying dampness, and killing insects to alleviate itching.Huanglian contains berberine, palmatine, berberine alkaloid, and epiberberine, with berberine and berberine alkaloid being the main bacteriostatic components. Berberine can inhibit the formation of C. albicans biofilms by downregulating the expression levels of EFG1, HWP1, ALS1, and ECE1 genes. Sanbaicao's main active ingredient, Sauchinone, exhibits anti-inflammatory effects by inhibiting NF-κB activity and reducing TNF-α expression in macrophages. Daqingye's effective antibacterial component, indirubin, can effectively inhibit the formation of Candida albicans mixed biofilms. Jiguanghua's main component, kaempferol, has strong anti-inflammatory effects by regulating the activity of pro-inflammatory enzymes and the expression of inflammation-related genes. The primary antibacterial active ingredient in Dingxiang is eugenol, which can disrupt the fungal cell membrane.

In summary, we have thoroughly separated and characterized the active substances in KBN lotion through UPLC-Q-TOF/MS analysis. On the other hand, I confirm that the revised manuscript has incorporated all necessary changes, with the modifications highlighted in red for clarity. The antimicrobial activity assays conducted in this study strictly adhered to the CLSI M27-A3 guidelines, ensuring that the methodologies used conform to recognized standards for evaluating antifungal susceptibility. We hope these revisions and additions address the previous concerns.
